# Proteomic Analysis of Serum and Cerebrospinal Fluid in Children with Encephalopathy Associated with Human Betaherpesvirus 6B

**DOI:** 10.1093/ofid/ofag095

**Published:** 2026-02-24

**Authors:** Yoshiki Kawamura, Hisateru Yamaguchi, Tomoki Nishioka, Mao Kiribuchi, Ayano Yun, Hiroki Miura, Yotaro Kondo, Masato Itano, Yuki Higashimoto, Masaru Ihira, Jun-ichi Kawada, Tetsushi Yoshikawa

**Affiliations:** Department of Pediatrics, Fujita Health University School of Medicine, Toyoake, Japan; Department of Pediatrics, Fujita Health University Okazaki Medical Center, Okazaki, Japan; Department of Medical Technology, School of Nursing and Medical Care, Yokkaichi Nursing and Medical Care University, Yokkaichi, Japan; Biomedical Molecular Sciences, Fujita Health University School of Medicine, Toyoake, Japan; Division of Cell Biology, International Center for Brain Science, Fujita Health University, Toyoake, Japan; Department of Pediatrics, Fujita Health University School of Medicine, Toyoake, Japan; Department of Pediatrics, Fujita Health University School of Medicine, Toyoake, Japan; Department of Pediatrics, Fujita Health University School of Medicine, Toyoake, Japan; Department of Pediatrics, Fujita Health University School of Medicine, Toyoake, Japan; Department of Pediatrics, Fujita Health University School of Medicine, Toyoake, Japan; Department of Pediatrics, Fujita Health University Okazaki Medical Center, Okazaki, Japan; Department of Clinical Microbiology, Fujita Health University School of Medical Sciences, Toyoake, Japan; Department of Clinical Science for Biological Monitoring, Fujita Health University School of Medical Sciences, Toyoake, Japan; Department of Pediatrics, Fujita Health University School of Medicine, Toyoake, Japan; Department of Pediatrics, Fujita Health University School of Medicine, Toyoake, Japan

**Keywords:** encephalitis, encephalopathy, human herpesvirus 6B, glycolysis, MARCKS

## Abstract

**Background:**

Exanthem subitum (ES), a benign febrile exanthematous disease, is caused by primary human betaherpesvirus 6B (HHV-6B) infection. It may cause neurological complications, including complex febrile seizures (cFS), acute encephalopathy with biphasic seizures, and late reduced diffusion (AESD). cFS resolves spontaneously; however, AESD can pose severe sequelae. We aimed to elucidate AESD pathogenesis using a proteomic analysis.

**Methods:**

Using liquid chromatography-tandem mass spectrometry (LC-MS/MS), serum and cerebrospinal fluid (CSF) protein profiles were compared between patients with AESD and those with cFS (*n* = 3 or 4 per group). Metascape was used for enrichment analysis, and the selected proteins were validated using a large sample via enzyme-linked immunosorbent assay (ELISA).

**Results:**

A total of 698 proteins were identified across all serum and CSF samples using LC-MS/MS. Nineteen serum proteins were differentially expressed in AESD and cFS during the acute phase. The glycolytic pathway was upregulated in AESD. Myristoylated alanine-rich C kinase substrate (MARCKS) and Golgi membrane protein 1 (GOLM1) were selected for validation using ELISA. Both proteins were upregulated during the acute phase (*n* = 11) compared with the convalescent phase (*n* = 21) in AESD (MARCKS, *P* = .016; GOLM1, *P* < .001). MARCKS during the acute phase was also upregulated in AESD compared with that in uncomplicated ES (*n* = 15) (*P* = .015). In CSF, 38 proteins were differentially expressed between AESD and cFS during the acute phase. Cholesteryl ester transfer protein in the CSF of patients with AESD was upregulated; however, this could not be validated using ELISA.

**Conclusions:**

Glycolysis and MARCKS pathways might be involved in HHV-6B-associated AESD pathogenesis.

## BACKGROUND

Human herpes betaherpesvirus 6B (HHV-6B) is a ubiquitous virus, with approximately 90% of Japanese children experiencing primary infection by 2 years of age. Exanthema subitum (ES) is a benign febrile exanthematous disease caused by primary HHV-6B infection [1, 2]. Owing to its relatively high incidence and some patients experiencing severe complications, central nervous system (CNS) involvement, such as febrile seizures (FS) and encephalitis, has become a clinically important issue [3, 4].

Complex febrile seizures (cFS) is characterized by focal features, a duration >15 minutes, or multiple seizures occurring within 24 hours, and is associated with a high risk of recurrence and a greater likelihood of developing subsequent afebrile seizures [5]. Reportedly, the incidence of cFS is significantly higher in patients with primary HHV-6B infection (48.4%) compared with those infected with other pathogens [6, 7]. Primary HHV-6B infection is the second most common cause of pediatric acute encephalopathy (16.5%) after influenza virus infection (17.4%) in Japan [8, 9] and represents a significant disease burden, as approximately half of the affected patients suffer from severe neurological sequelae [10].

Pediatric acute encephalopathy is classified into various clinicoradiological syndromes. Acute encephalopathy with biphasic seizures and late reduced diffusion (AESD) is the most common syndrome (66.3%) in primary HHV-6-infected patients, followed by mild encephalopathy with reversible splenial lesion (4.3%) and acute necrotizing encephalopathy (2.7%) [9]. AESD is characterized by biphasic seizures and is difficult to distinguish from cFS, which does not require specific treatment during the first febrile convulsion episode. Thus, elucidating the differences in pathogenesis between the two complications is important, as well as discovering reliable biomarkers to distinguish between them.

Analysis of cytokines, chemokines, and viral DNA loads in the cerebrospinal fluid (CSF) shows that HHV-6B does not directly invade the CNS during primary infection. Instead, host immune responses play a key role in development of acute encephalopathy [4, 11, 12]. However, only a limited number of biomarkers have been analyzed in previous studies [4, 11–13], and a more comprehensive biomarker analysis is needed.

Quantitative proteomic profiling using mass spectrometry (MS) has been used to elucidate immune response variations in various diseases, including infectious diseases [14–17]. Through the identification of key proteins involved in biological pathways, such as pathogen invasion and host immune defense, these studies have advanced our understanding of disease mechanisms, and potential targets for therapeutic interventions have been suggested.

In this study, we performed a comprehensive proteomic analysis using serum and CSF from patients with AESD and cFS due to primary HHV-6B infection, as well as from healthy controls (HC), using MS-based approaches. Candidate proteins associated with AESD were validated using enzyme-linked immunosorbent assay (ELISA).

## METHODS

### Patients and Samples

Serum and CSF samples were collected from Japanese patients virologically diagnosed with primary HHV-6B infection complicated by either AESD or cFS in our department. CSF and blood samples were collected concurrently. In addition, the following two control groups were enrolled: (1) the uncomplicated ES group, comprising children with primary HHV-6B infection without seizures or neurological complications and (2) the HC group, comprising age-matched children without HHV-6B infection or clinical symptoms and confirmed negative for HHV-6B DNA by polymerase chain reaction. The HC group was included to identify the unique proteomic signature of AESD in liquid chromatography-tandem mass spectrometry (LC-MS/MS) analysis, whereas the uncomplicated ES group was included as a control for AESD in the ELISA. The clinical characteristics of the groups whose serum or CSF samples were used for LC-MS/MS and ELISA are shown in Supplementary Tables 1 and 2 and Tables 1 and 2, respectively. There were no statistically significant differences in age or sex among the groups (AESD, cFS, uncomplicated ES, and HC). Informed consent was obtained using an opt-out method. This study was approved by the Ethical Review Board of Human Studies of Fujita Health University (accession number: HM23-380).

**Table 1.** Characteristics of Patients Whose Serum Proteins Were Measured by ELISA

Group	AESD			cFS	ES	*P*-value
Phase (*n*)	Early (11)	Late (26)	Convalescent (21)	Acute (26)	Acute (15)	
Age (years) (Median/IQR)	0.92/0.75–1	1/0.83–1	0.92/0.92–1	1/0.98–1	1/0.75–1	.242
Sex (male/female)	4/7	15/11	12/9	15/11	9/6	.681
Days of specimens collection^a^ (Median/IQR)	1/1–2	5/4–6	19/13–30	2/1–2	3/2–3	<.01
HHV-6B DNA load (copies/μgDNA) (Median/IQR)	33 595/ 5580–52 375	1475/ 0–12 088	0/ 0–0	45 650/ 17 275–72 625	26 289/ 10 063–99 263	<.01 (.670^b^)

^a^The first day of fever was defined as day 1.

^b^Comparison among AESD early, cFS acute, and ES acute.

AESD, acute encephalopathy with biphasic seizures and late reduced diffusion; cFS, complex febrile seizures; ELISA, enzyme-linked immunosorbent assay; ES, exanthema subitum; HHV-6B, human herpesvirus 6B; IQR, interquartile range.

**Table 2.** Characteristics of Patients Whose Proteins in CSF Were Measured by ELISA

Group	AESD		cFS	*P*-value
Phase (*n*)	Early (12)	Late (15)	Acute (35)	
Age (years) (Median/IQR)	0.96/0.77–1	0.92/0.75–1	1/0.92–1	.108
Sex (male/female)	6/6	5/10	21/14	.202
Days of specimen collection^a^ (Median/IQR)	1/1–2	5/4–6	2/2-2	<.001
HHV-6B DNA load (copies/μgDNA) (Median/IQR)	0/0–0	0/0–3550	0/0–0	.020

^a^The first day of fever was defined as day 1.

AESD, acute encephalopathy with biphasic seizures and late reduced diffusion; cFS, complex febrile seizures; ELISA, enzyme-linked immunosorbent assay; HC, healthy controls.; IQR, interquartile range.

Primary HHV-6 infection was diagnosed based on viral isolation from peripheral blood mononuclear cells using cord blood mononuclear cells, as previously described, or the detection of viral DNA in the serum and serological analysis [18, 19]. AESD was diagnosed using clinical and radiological criteria [8, 20], which included: (1) a biphasic seizure pattern, with the first seizure occurring during the febrile period; (2) a second seizure occurring on days 4–7 of illness without fever; and (3) characteristic neuroimaging findings, including reduced diffusion in white matter on diffusion-weighted imaging. Early seizures were defined as those that occurred during the febrile period (AESD-early), whereas late seizures were defined as the second phase of seizures on days 4–7 of illness (AESD-late) and convalescence as the period after day 10 (AESD-convalescent). cFS were defined as seizures with focal features (seizure activity limited to one side of the body rather than generalized convulsions), duration >15 minutes, or recurrence within 24 hours, occurring during the febrile period without evidence of biphasic seizures or characteristic white matter involvement on imaging [5].

### Sample Preparation for Mass Spectroscopy Analysis

Serum samples were processed using High-Select Top 14 Abundant Protein Depletion Mini Columns (Thermo Fisher Scientific, Rockford, IL, USA) to remove 14 highly abundant human proteins [albumin, immunoglobulin (Ig)A, IgD, IgE, IgG, IgG (light chains), IgM, α1-acid glycoprotein, α1-antitrypsin, α2-macroglobulin, apolipoprotein A1, fibrinogen, haptoglobin, and transferrin], following the manufacturer's instruction. Approximately 5 μg protein of the serum samples were digested with trypsin using single-pot solid-phase-enhanced sample preparation (SP3) protocol [21]. CSF was digested with trypsin without using the High-Select Top 14 Abundant Protein Depletion Mini Columns or the SP3 protocol.

### LC-MS/MS Analysis

To identify AESD-associated proteins, LC-MS/MS analysis was performed using three or four samples from each group (Supplementary Tables 1 and 2) as previously described [22]. Briefly, samples were analyzed using a LC system (EASY-nLC 1000; Thermo Fisher Scientific, Rockford, IL, USA) coupled with an MS system (Orbitrap Fusion Tribrid Mass Spectrometer; Thermo Fisher Scientific, Rockford, IL, USA). Each patient sample was analyzed individually. The LC and MS acquisition conditions have been previously described [23, 24]. Peptide ions were detected using the Xcalibur software (version 4.1; Thermo Fisher Scientific, Rockford, IL, USA). MS/MS searches were conducted using MASCOT (version 2.6.2; Matrix Science, London, UK) and SEQUEST HT algorithms against the SwissProt *Homo sapiens* database (2022-12-14) via Proteome Discoverer (PD, version 2.4.1.15; Thermo Fisher Scientific, Rockford, IL, USA).

Principal component analysis (PCA) was subsequently conducted with PD to evaluate global protein expression patterns across groups [25]. Enrichment analysis of the differentially expressed proteins was performed using Metascape (version 3.5) [26]. Enriched terms were statistically identified among the gene ontology/Kyoto Encyclopedia of Genes and Genomes terms, canonical pathways, and hallmark gene sets. Accumulative hypergeometric *P*-values and enrichment factors were calculated and used for filtering. The remaining significant terms were subsequently hierarchically clustered into a tree based on the kappa statistical similarities among their gene memberships. A kappa score of 0.3 was used as the threshold to cast the tree into term clusters. The terms with the best *P*-value within each cluster were selected as representative terms.

### Experimental Validation of Candidate Protein Biomarkers

To validate the selected AESD-associated proteins identified using the LC-MS/MS, their expression levels in serum and CSF were examined in a larger number of samples using ELISA. The following commercial kits were used following the manufacturer's instructions: human myristoylated alanine-rich C kinase substrate (MARCKS) ELISA Kit (MyBiosource, San Diego, CA, USA), Golgi membrane protein 1 (GOLM1) Sandwich ELISA Kit (Proteintech, Rosemont, IL, USA), and human cholesteryl ester transfer protein (CETP) ELISA kit (Novus Biologicals, Centennial, CO, USA).

### Statistical Analysis

Label-free quantification was performed using PD, and statistical comparisons were performed using the Perseus software (version 1.6.15.0; Max Planck Institute of Biochemistry, Planegg, Germany) [27]. Proteins with a fold change >1.5 or <0.67 (equivalent to 2/3) and an adjusted *P-*value <.05 (Student's *t* test with correction for multiple comparisons) were considered statistically significant. Spearman's rank correlation coefficient (Spearman's ρ) was used to assess the relationship between protein expression levels in serum and CSF. Proteins of interest were then selected from those showing statistically significant differences in expression. Categorical variables, such as sex, were compared among the groups using Fisher's exact test. Continuous variables, including age, days of specimen collection, HHV-6B DNA load, and protein concentrations measured by ELISA, were compared among the groups using the Kruskal–Wallis. The Steel–Dwass test (JMP 14.2.2.0; SAS Institute, Cary, NC, USA) when a significant difference was detected.

## RESULTS

### Protein Expressions in Serum and Cerebrospinal Fluids

To comprehensively analyze protein expression, LC-MS/MS was initially performed on serum and CSF samples obtained from six patient groups: AESD-early, AESD-late, AESD-convalescent, acute-phase cFS (cFS-acute), convalescent-phase cFS (cFS-convalescent), and HC (Supplementary Tables 1 and 2), each consisting of three or four patients. CSF was digested with trypsin without using the High-Select Top 14 Abundant Protein Depletion Mini Columns and SP3 protocol because this treatment reduced the number of proteins identified via LC-MS/MS (Supplementary Figure 1). Overall, 698 proteins were identified in all serum and CSF samples for subsequent quantification: 459 in serum and 498 in CSF. PCA visualization was conducted for these proteins (Supplementary Figure 2). The PCA of all samples revealed distinct clustering between the serum and CSF samples. Based on this separation, subsequent analyses were performed independently for the serum and CSF. In the serum PCA, the AESD-early samples were plotted in a region distinct from the AESD-convalescent and HC samples (Supplementary Figure 3). PCA of the CSF also varied among each phase of AESD and cFS-acute (Supplementary Figure 4).

### Identification of AESD-related Proteins

Proteins that were significantly different between AESD-acute and AESD-late, AESD-convalescent, cFS-acute, and HC were identified to determine AESD diagnostic markers during the acute phase of the disease and elucidate the pathogenesis of AESD. The number of differentially expressed proteins in AESD-early compared with those in AESD-late, AESD-convalescent, cFS-acute, and HC in the serum and CSF is shown in Supplementary Table 3. The specific proteins differentially expressed in the serum from the AESD-early group compared with those in the AESD-late, AESD-convalescent, and HC groups are listed in Supplementary Tables 4–6. Differentiating between AESD and cFS is often challenging in clinical practice. Nineteen serum proteins were differentially expressed in the AESD-early group compared with those in the cFS-acute group are shown in Table 3. No correlation was observed between serum and CSF protein levels for any protein. Among these, MARCKS had the lowest *P*-value (*P* = .001) among the proteins upregulated in the AESD-early compared with those in cFS-acute (Table 3). Furthermore, GOLM1 was consistently upregulated in the AESD-early group compared with those in all other groups (Supplementary Figure 5).

**Table 3.** Differentially Expressed Serum Proteins in Patients with AESD Early Compared with Those with cFS Acute and Their Correlation Between Serum and CSF

	Student's *t* test		Spearman's Rank Correlation Coefficient	
Proteins	Log_2_ Fold Change	Adjusted *P*-value	Spearman's ρ	*P*-value
Fructose-bisphosphate aldolase B	4.836	.012	N/A	N/A
Tropomyosin alpha-1 chain	3.627	.002	−0.086	.872
Talin-1	3.051	.025	N/A	N/A
Collagen alpha-1(XVIII) chain	2.972	.028	−0.200	.704
**Myristoylated alanine-rich C-kinase substrate (MARCKS)**	2.590	.001	0.086	.872
Pleckstrin	2.420	.034	0.314	.544
Moesin	2.199	.015	N/A	N/A
**Golgi membrane protein 1 (GOLM1)**	1.642	.013	0.543	.266
Calreticulin	1.399	.039	−0.371	.468
Endoplasmic reticulum chaperone BiP	1.228	.006	−0.257	.623
CD109 antigen	0.792	.039	N/A	N/A
GDH/6PGL endoplasmic bifunctional protein	0.690	.033	N/A	N/A
Golgi-associated plant pathogenesis-related protein 1	0.641	.009	0.829	.042
Serpin A11	−0.899	.028	N/A	N/A
Immunoglobulin heavy constant mu	−1.025	.005	−0.600	.208
Immunoglobulin kappa variable 4-1	−1.074	.009	0.429	.397
Immunoglobulin heavy variable 3-15	−1.081	.045	−0.086	.872
Spondin-1	−1.328	.045	N/A	N/A
Immunoglobulin J chain	−2.678	.035	−0.371	.468

Proteins in bold were verified by enzyme-linked immunosorbent assay.

AESD, acute encephalopathy with biphasic seizures and late reduced diffusion; cFS, complex febrile seizures; CSF, cerebrospinal fluid; N/A, not applicable.

The differentially expressed proteins between AESD-early and AESD-late or AESD-convalescent CSF are listed in Supplementary Tables 7 and 8. Thirty-eight CSF proteins were differentially expressed between the AESD-early and cFS-acute groups, and their correlations with serum levels are shown in Table 4. Among the upregulated proteins in the AESD-early group, CETP showed the lowest *P*-value.

**Table 4.** Differentially Expressed Proteins of CSF in Patients with AESD Early Compared to cFS Acute and Their Correlation Between Serum and CSF

	Student's *t* test		Spearman's Rank Correlation Coefficient	
Proteins	Log_2_ Fold Change	Adjusted *P*-value	Spearman's ρ	*P*-value
Hemoglobin subunit gamma-1	4.27	.0138	0.771	.072
Serum amyloid P-component	3.61	.0209	−0.143	.787
Hemoglobin subunit alpha	3.12	.0489	−0.200	.704
Prenylcysteine oxidase 1	2.61	.0084	0.086	.872
Thyroxine-binding globulin	1.87	.0125	−0.143	.787
**Cholesteryl ester transfer protein (CETP)**	1.56	.0032	−0.257	.623
Immunoglobulin heavy constant mu	1.47	.0433	−0.600	.208
Gelsolin	−0.64	.0278	N/A	N/A
Selenoprotein P	−0.65	.0236	0.314	.544
Prosaposin	−0.65	.0493	0.257	.623
Tripeptidyl-peptidase 1	−0.81	.0107	0.714	.111
Immunoglobulin heavy constant alpha 1	−0.85	.0226	−0.714	.111
Immunoglobulin superfamily member 8	−0.88	.0264	0.812	.050
Sulfhydryl oxidase 1	−0.97	.0231	0.486	.329
Heparin cofactor 2	−1.04	.0184	0.314	.544
Mimecan	−1.14	.0291	0.429	.397
Carboxypeptidase E	−1.15	.0310	−0.257	.623
Monocyte differentiation antigen CD14	−1.15	.0311	0.143	.787
Complement C1q subcomponent subunit A	−1.19	.0411	0.725	.103
Glutathione peroxidase 3	−1.21	.0240	−0.086	.872
Scrapie-responsive protein 1	−1.21	.0447	N/A	N/A
Ganglioside GM2 activator	−1.23	.0283	N/A	N/A
Complement component C9	−1.29	.0330	0.429	.397
Tissue alpha-L-fucosidase	−1.42	.0267	−0.600	.208
Protein-L-isoaspartate(D-aspartate) O-methyltransferase	−1.69	.0434	N/A	N/A
Transcobalamin-2	−1.71	.0133	N/A	N/A
Cell adhesion molecule 2	−1.76	.0153	N/A	N/A
14-3-3 protein eta	−1.78	.0075	N/A	N/A
Flotillin-1	−1.83	.0358	−0.143	.787
Phosphatidylcholine-sterol acyltransferase	−1.85	.0241	0.029	.957
Beta-hexosaminidase subunit beta	−1.92	.0187	N/A	N/A
Probable serine carboxypeptidase CPVL	−1.92	.0259	N/A	N/A
Phospholipid transfer protein	−2.07	.0244	0.543	.266
Cerebellin-3	−2.09	.0174	N/A	N/A
Glyceraldehyde-3-phosphate dehydrogenase	−2.22	.0231	−0.543	.266
Inter-alpha-trypsin inhibitor heavy chain H5	−2.38	.0408	N/A	N/A
Ubiquitin-60S ribosomal protein L40	−2.74	.0297	−0.429	.397
Protein Z-dependent protease inhibitor	−3.34	.0124	−0.257	.623

Proteins in bold were verified by enzyme-linked immunosorbent assay.

AESD, acute encephalopathy with biphasic seizures and late reduced diffusion; cFS, complex febrile seizures; N/A, not applicable.

### Enrichment Analysis

**Figure 1.**
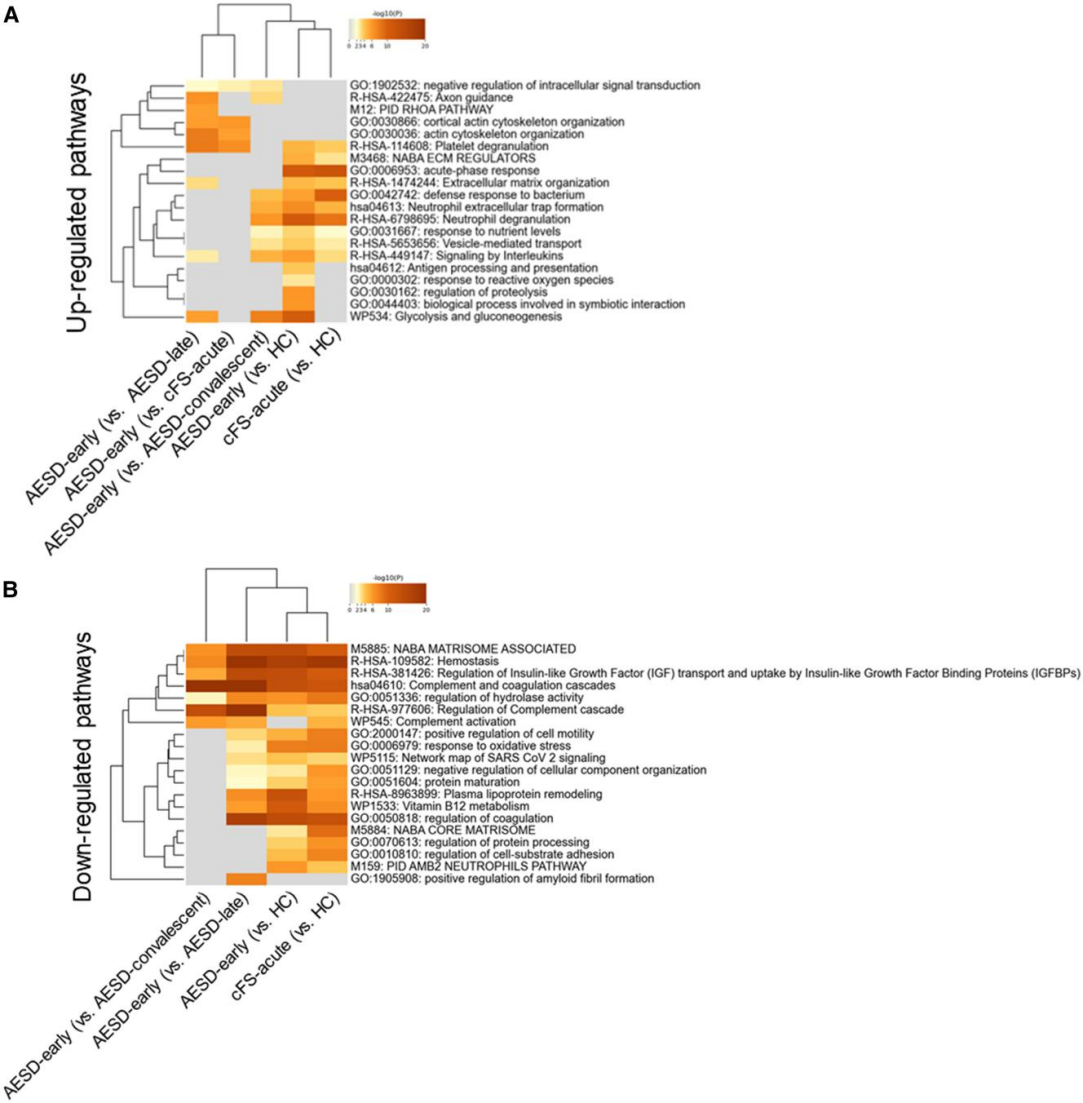
Heat map of enrichment in serum of patients with human herpesvirus 6B (HHV-6B)-associated-acute encephalopathy with biphasic seizures and late reduced diffusion (AESD), patients with HHV-6B-associated-complex febrile seizures (cFS), and healthy controls (HC). The dendrogram includes terms that were upregulated (*A*) and downregulated (*B*) in the serum of patients with AESD-early or cFS-acute. Enriched terms were statistically identified among the gene ontology/Kyoto Encyclopedia of Genes and Genomes terms, canonical pathways, and hallmark gene sets. The heatmap cells are colored according to their *P*-values, and white cells indicate a lack of enrichment for that term in the corresponding list of genes. AESD-early, seizures within the febrile period; AESD-late, the second phase of seizures; cFS-acute, acute-phase complex febrile seizures; AESD-convalescent, the period after day 10; HC, Healthy controls.

**Figure 2.**
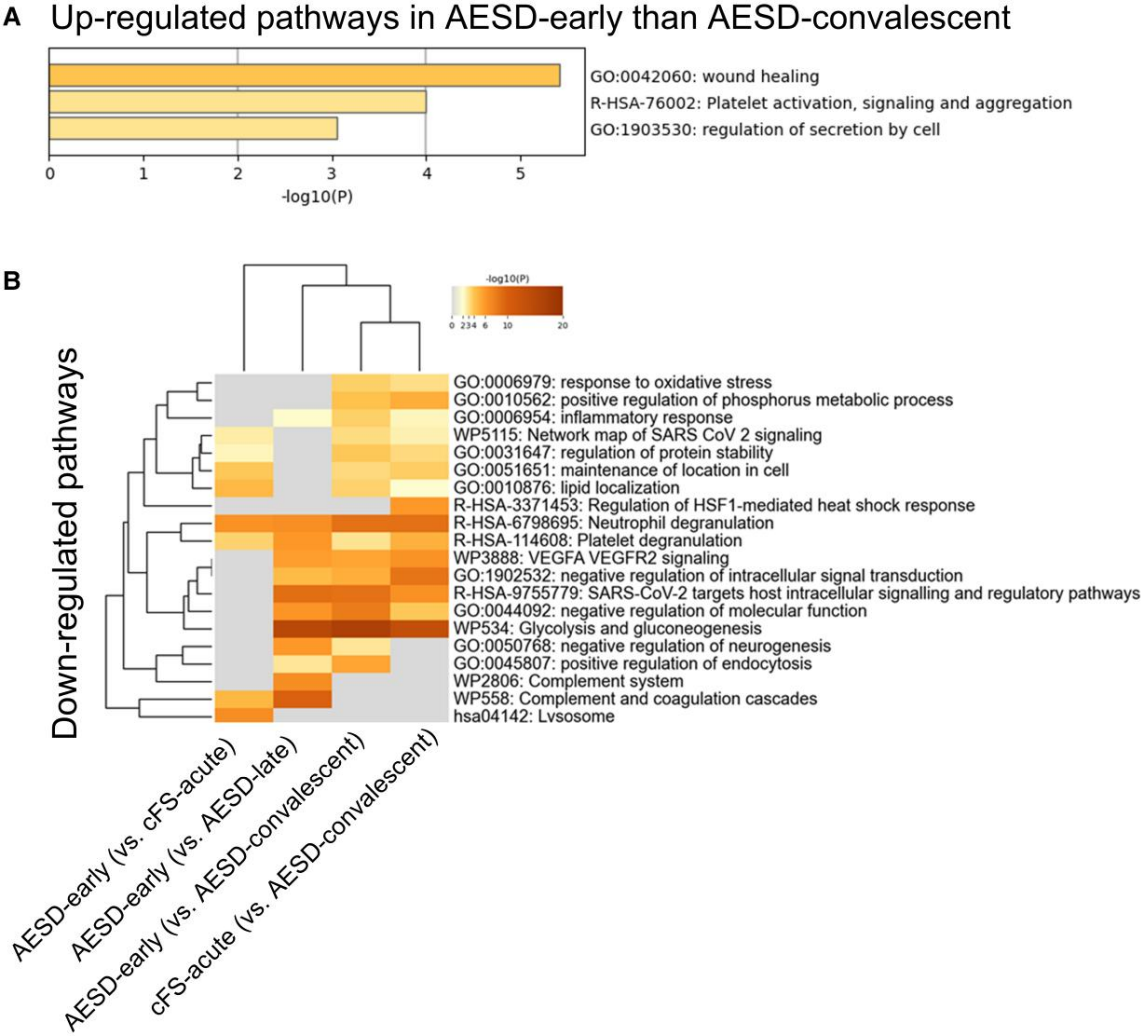
Heat map of enrichment in cerebrospinal fluid (CSF) of patients with human herpesvirus 6B (HHV-6B)-associated-acute encephalopathy with biphasic seizures and late reduced diffusion (AESD), patients with HHV-6B-associated-complex febrile seizures (cFS) and healthy controls (HC). The dendrogram included terms that were upregulated (*A*) and downregulated (*B*) in the cerebrospinal fluids of patients with AESD-early or cFS-acute. Enriched terms were statistically identified among the gene ontology/Kyoto Encyclopedia of Genes and Genomes terms, canonical pathways, and hallmark gene sets. The heatmap cells are colored according to their *P*-values, and white cells indicate a lack of enrichment for that term in the corresponding list of genes. AESD-early, seizures within the febrile period; AESD-late, the second phase of seizures; cFS-acute, acute-phase complex febrile seizures; AESD-convalescent, the period after day 10.

The results of the pathway enrichment analysis of differentially expressed proteins in serum and CSF using Metascape are shown in Figures 1 and 2, respectively. The number of differentially expressed proteins used in each analysis is shown in Supplementary Table 3. In the serum, immune-related pathways, such as the acute-phase response, neutrophil degranulation, and interleukin signaling, were upregulated in AESD-early and cFS-acute samples compared with those in HC (Figure 1*A*). Cytoskeletal pathways, including cortical actin cytoskeleton organization and general actin cytoskeleton organization, were upregulated in AESD-early samples compared with those in the AESD-late and cFS-acute samples. In addition, the glycolysis/gluconeogenesis pathway was significantly upregulated in the AESD-early group compared with that in the AESD-late, AESD-convalescent, and cFS-acute groups. Conversely, complement-related pathways were downregulated in the AESD-early and cFS-acute groups relative to those in the HC group (Figure 1*B*). No specific pathways were uniquely downregulated in the AESD-early group compared with those in the other disease groups.

In the CSF, pathways of wound healing, platelet activation, signaling, aggregation, and regulation of secretion by cells were upregulated in the AESD-early group compared with those in the AESD-convalescent group (Figure 2*A*). Meanwhile, immune-related pathways, including the inflammatory response or neutrophil degranulation, negative regulation of neurogenesis, and glycolysis/gluconeogenesis, were downregulated in the AESD-early and cFS-acute groups compared with those in the AESD-convalescent group (Figure 2*B*). Additionally, pathways such as neutrophil and platelet degranulation were downregulated in the AESD-early compared with the cFS-acute groups (Figure 2*B*).

### Validation of Candidate Protein Biomarkers Using ELISA

**Figure 3.**
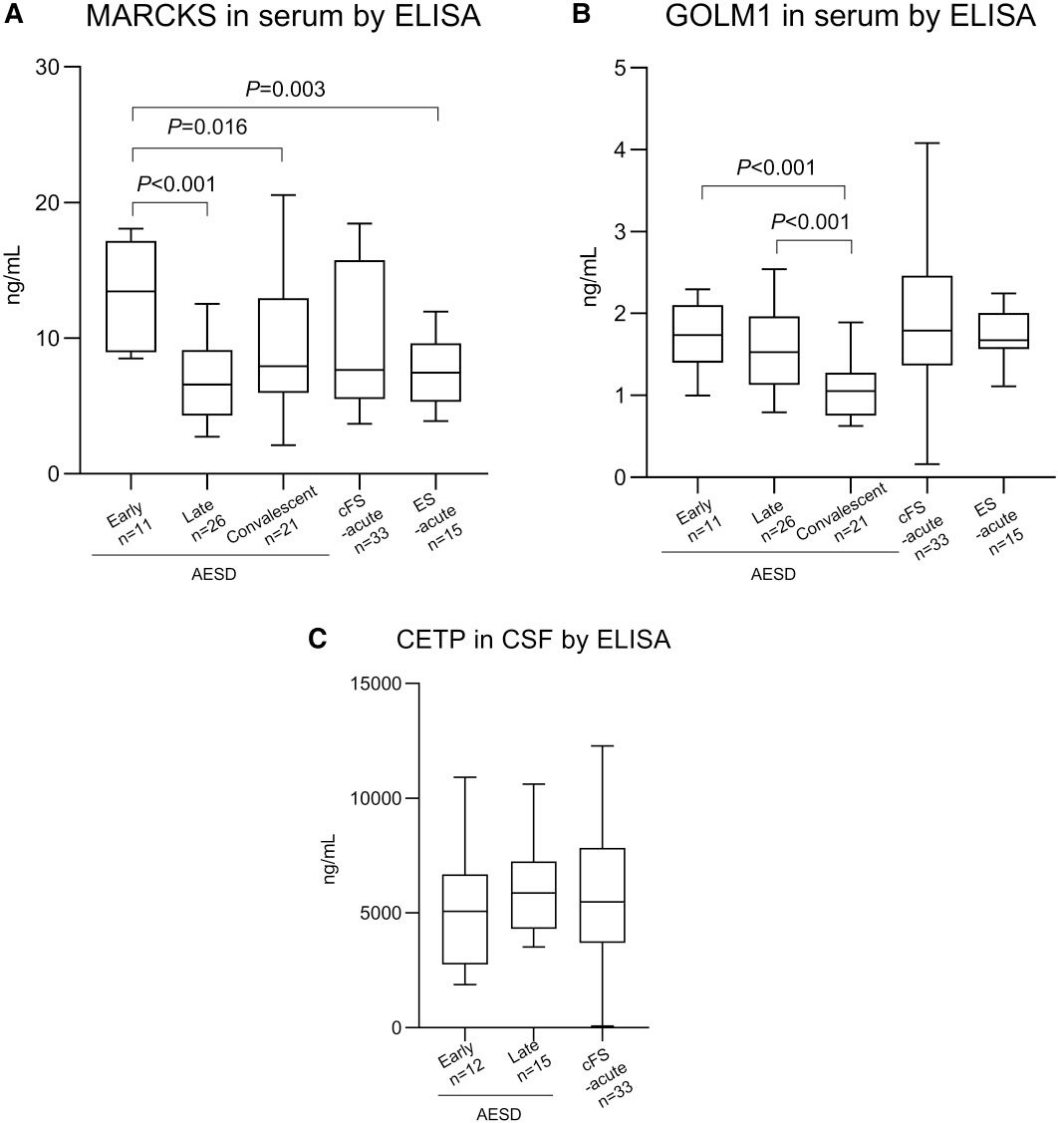
Serum and cerebrospinal fluid (CSF) concentrations of proteins in patients with human herpesvirus 6B (HHV-6B)-associated-acute encephalopathy with biphasic seizures and late reduced diffusion (AESD), patients with HHV-6B-associated-complex febrile seizures (cFS), and patients with exanthema subitum (ES). Serum concentrations of myristoylated alanine-rich c-kinase substrates (MARCKS, *A*), Golgi membrane protein 1 (GOLM1, *B*), and cholesteryl ester transfer protein (CETP, *C*) are shown. The centerlines of the boxes denote the median values, whereas the boxes contain the 25th and 75th percentiles. Whiskers extend from the top and bottom of the box, representing a 1.5 times interquartile range.

To validate candidate protein biomarkers for diagnosing AESD in the early seizure phase, ELISAs were performed for MARCKS and GOLM1 using serum and for CETP using CSF (Figure 3) obtained from a separate patient cohort. Serum samples were collected from 11 patients with AESD-early, 26 patients with AESD-late, 21 patients with AESD-convalescent, 26 patients with cFS-acute, and 15 patients with uncomplicated ES (Table 1). CSF samples were obtained from 12 patients with AESD-early, 15 with AESD-late, and 35 with cFS-acute (Table 2). The selected protein concentrations were initially compared between the AESD phases. Additionally, the selected protein concentrations in the AESD-early stages were compared with those in the cFS-acute and ES-acute stages (Figure 3). Serum MARCKS levels were significantly higher in the AESD-early group than in the AESD-late (*P* < .001) and AESD-convalescent groups (*P* = .016) (Figure 3*A*). Moreover, these levels were significantly higher in the AESD-early group than in the ES group (*P* = .003) (Figure 3*A*). However, MARCKS levels were not significantly different between AESD-early and cFS-acute (Figure 3*A*). GOLM1 levels in the serum were significantly elevated in AESD-early compared with those in the AESD-convalescent (*P* < .001) (Figure 3*B*). However, levels were not significantly different among the AESD-early, cFS-acute, and ES groups (Figure 3*C*).

## DISCUSSION

In this study, LC-MS/MS analysis of serum and CSF was performed to investigate the pathogenesis of AESD caused by primary HHV-6B infection. Enrichment analysis of serum samples revealed the upregulation of immune-related pathways and downregulation of complement pathways in the AESD-early and cFS-acute groups. The complement system is involved in genetic susceptibility to epileptic seizures [28], suggesting that complement modulation may critically influence seizures caused by AESD and cFS following HHV-6B infection. Moreover, pathways related to cortical actin cytoskeleton organization were upregulated in the AESD-early group compared with those in the AESD-late and cFS-acute groups. Furthermore, excitotoxic neuronal injury has been proposed as a core mechanism of AESD pathogenesis [29], although no significant increase in serum or CSF glutamate/glutamine levels was observed in this study. Proteomic signatures suggest that neuronal damage contributes to AESD progression, although excitotoxicity has not been directly demonstrated. Therefore, early seizure control [30] and neuroprotective strategies, such as therapeutic hypothermia [31] may be critical for prevention.

The glycolysis and gluconeogenesis pathways were upregulated in patients with AESD-early compared with those in patients with AESD-convalescent and HC. Glycolysis is the anaerobic breakdown of glucose into lactate, while gluconeogenesis involves the regeneration of glucose from pyruvate or lactate. These metabolic pathways are reportedly associated with viral replication and immune responses in various types of viral infections, including poliovirus [32], flaviviruses [33, 34], and West Nile virus [35]. In addition, glycolysis contributes considerably to activating the immune and inflammatory responses; hence, it is a promising therapeutic target for viral infections [36]. Furthermore, *Herpes simplex* encephalitis was recently reported to alter glycolytic protein expression in CSF [37]. Collectively, these findings and the present data suggest that the early upregulation of glycolytic metabolism may be involved in the pathogenesis of AESD. Thus, supporting glucose metabolism during early seizures, such as appropriate oxygen administration to reverse anaerobic metabolic states [38], optimal glycemic control [39], and mitochondrial cocktails to rescue mitochondria, the critical sites for glucose metabolism [40] maybe an AESD treatment option.

In the CSF, platelet activation, signaling, and aggregation pathways were upregulated in patients with AESD-early compared with those in patients AESD-convalescent. Reportedly, elevated CSF platelet-derived growth factors may be involved in the pathogenesis of influenza-associated encephalopathy [41]. Therefore, the present data show that platelet-related mechanisms contribute to the pathogenesis of AESD caused by primary HHV-6B infection. Notably, in contrast to the serum, immune pathways such as inflammatory responses and neutrophil degranulation, as well as glycolysis/gluconeogenesis, were downregulated in the CSF of patients with acute AESD-early and cFS compared with those in patients with AESD-convalescent. As previously reported, cytokine profiles differ between serum and CSF in AESD [11], and different patterns of protein expression were demonstrated using PCA in this study. These findings suggest that maintaining immune balance and metabolic homeostasis in the serum and CSF may be essential for preventing AESD. Additionally, neurogenesis and complement pathways were downregulated in the CSF of AESD-early rats. In addition to immune responses, controlling complement dysregulation may be important for AESD treatment because complement components contribute to neurodevelopment and synaptic function, and their dysregulation results in neurodegeneration [42].

To validate the results of the proteomic profiling using LC-MS/MS, ELISAs were conducted for MARCKS and GOLM1 in the serum and CETP of the CSF. MARCKS levels were significantly elevated in AESD-early serum compared with those in AESD-late, AESD-convalescent, and uncomplicated ES. MARCKS is a membrane-associated protein that is expressed in many cell types, including macrophages. It is functionally implicated in specific biological processes, including the innate immune response, inflammatory response, cytokine production, and molecular functions such as extracellular ATP-gated cation channel activity, electron transfer activity, and oxidoreductase activity [43, 44]. MARCKS appears to be a key regulator of AESD, and inhibiting it might be beneficial for therapeutic or preventive interventions against the disease. However, MARCKS levels did not significantly differ between AESD-early and cFS-acute, indicating its limited utility as a diagnostic marker.

A major limitation of this study is that the enrichment analysis findings were not validated using additional experimental methods. To definitively determine the roles of pathways such as neuronal injury, glycolysis, immune activation, and complement in AESD pathogenesis, further validation using larger patient cohorts and targeted assays is required.

In conclusion, LC-MS/MS analysis of the serum and CSF of patients with AESD and primary HHV-6B infection revealed key pathways potentially involved in disease development, including immune activation, complement regulation, neuronal injury, and altered glucose metabolism. Modulation of these pathways may provide therapeutic avenues for preventing AESD and improving clinical outcomes. Further studies with larger sample sizes are required to confirm these findings.

## Supplementary Material

ofag095_Supplementary_Data

## Supplementary Data

Supplementary materials are available at *Open Forum Infectious Diseases* online. Consisting of data provided by the authors to benefit the reader, the posted materials are not copyedited and are the sole responsibility of the authors, so questions or comments should be addressed to the corresponding author.
