# Supplementary material for: Proteomic Analysis of Serum and Cerebrospinal Fluid in Children with Encephalopathy Associated with Human Betaherpesvirus 6B
Source: Open Forum Infect Dis. 2026 Feb 24;13(3):ofag095. doi: 10.1093/ofid/ofag095 (PMC12973172; doi:10.1093/ofid/ofag095)
Supplement: ofag095_Supplementary_Data [file ofag095_supplementary_data.zip › RevisedSupFigLegends.docx]

**Figure S1. Optimization of sample preparation for mass spectrometry (MS) analysis**

To determine the optimal treatment for serum and cerebrospinal fluid (CSF) samples for mass spectroscopy (MS) analysis, specimens were either untreated or processed using High-Select Top 14 Abundant Protein Depletion Mini Columns (Top 14) or High-Select HSA/Immunoglobulin Depletion Resin (HSA/Ig) (Thermo Fisher Scientific, Rockford, IL, USA). The samples were subsequently subjected to liquid chromatography-tandem MS, and the number of detected proteins was quantified.

HSA/Ig, HSA/Immunoglobulin Depletion Resin

**Figure S2.** **Principal component analysis (PCA) of serum and cerebrospinal fluid (CSF)**

Principal component analysis (PCA) was performed on the proteomic data derived from serum (orange) and CSF (blue) samples to evaluate group-wise separation. Principal components 1 and 2 (PC1 and PC2) are plotted, accounting for 40.9% and 11.7% of the total variance, respectively. PCA was conducted using Proteome Discoverer (PD, version 2.4.1.15; Thermo Fisher Scientific, Rockford, IL, USA).

PC1, principal component 1; PC2, principal component 2

**Figure S3. Principal component analysis (PCA) of serum of patients with human herpesvirus 6B (HHV-6B)-associated-acute encephalopathy with biphasic seizures and late reduced diffusion (AESD), patients with HHV-6B-associated-complex febrile seizures (cFS), and healthy controls**

Principal component analysis (PCA) was performed on proteomic data derived from serum samples to evaluate group-wise separation among patients with HHV-6B-AESD-early (red), HHV-6B-AESD-late (pink), HHV-6B-AESD-convalescent (orange), HHV-6B-cFS-acute (light blue), HHV-6B-cFS-convalescent (green), and healthy controls (blue). Principal components 1 and 2 (PC1 and PC2) are plotted, using the17.7% and 15.2% of the total variance in the serum, respectively. PCA was conducted using Proteome Discoverer (PD, version 2.4.1.15; Thermo Fisher Scientific, Rockford, IL, USA). AESD-early, seizures within the febrile period; AESD-late, the second phase of seizures; cFS-acute, acute-phase complex febrile seizures; AESD-convalescent, the period after day 10.

**Figure S4. Principal component analysis (PCA) of cerebrospinal fluids (CSF) of patients with human herpesvirus 6B (HHV-6B)-associated-acute encephalopathy with biphasic seizures and late reduced diffusion (AESD) and patients with HHV-6B-associated-complex febrile seizures (cFS)**

Principal component analysis (PCA) was performed on proteomic data derived from cerebrospinal fluids (CSF) samples to evaluate group-wise separation among patients with HHV-6B-AESD-early (red), HHV-6B-AESD-late (pink), HHV-6B-AESD-convalescent (orange), and HHV-6B-cFS-acute (light blue). Principal components 1 and 2 (PC1 and PC2) accounted for 28.7% and 18.8% of the total variance in the serum, respectively. PCA was conducted using Proteome Discoverer (PD, version 2.4.1.15; Thermo Fisher Scientific, Rockford, IL, USA). AESD-early, seizures within the febrile period; AESD-late, the second phase of seizures; cFS-acute, acute-phase complex febrile seizures; AESD-convalescent, the period after day 10.

**Figure S5. Serum and cerebrospinal fluid (CSF) data measured by liquid chromatography-tandem mass spectrometry (LC-MS/MS) in patients with human herpesvirus 6B (HHV-6B)-associated acute encephalopathy with biphasic seizures and late reduced diffusion (AESD), patients with HHV-6B-associated complex febrile seizures (cFS), and healthy controls (HC)**

The centerlines of the boxes denote the median values, whereas the boxes represent the 25th and 75th percentiles. Whiskers extend from the top and bottom of the boxes, representing 1.5 times the interquartile range.

AESD-early, seizures within the febrile period; AESD-late, the second phase of seizures; AESD-convalescent, the period after day 10; cFS-acute, acute-phase complex febrile seizures; cFS-convalescent, convalescent phase cFS; CETP, cholesterol ester transfer protein; MARCKS, myristoylated alanine-rich C-kinase substrates; GOLM1, Golgi membrane protein 1.
