# Supplementary material for: Proteomic Analysis of Serum and Cerebrospinal Fluid in Children with Encephalopathy Associated with Human Betaherpesvirus 6B
Source: Open Forum Infect Dis. 2026 Feb 24;13(3):ofag095. doi: 10.1093/ofid/ofag095 (PMC12973172; doi:10.1093/ofid/ofag095)
Supplement: ofag095_Supplementary_Data [file ofag095_supplementary_data.zip › RevisedSupTableHHV-6AESDcFSSerumCSFProteomics.docx]

| **Sup. Table 1. Patient characteristics of the serum for which LC-MS/MS was performed** | | | | | | | | |
| --- | --- | --- | --- | --- | --- | --- | --- | --- |
| **Group** | **AESD** | | |  | **cFS** | |  | **HC** |
| Phase (n) | Early (4) | Late (4) | Convalescent (4) |  | Acute (4) | Convalescent (4) |  | (4) |
| Age (year) (Range) | 0.67−1 | 0.67−1 | 0.67−1 |  | 0.75−1 | 0.75−2 |  | 1−1 |
| Sex (Male/Female) | 2/2 | 2/2 | 2/2 |  | 2/2 | 3/1 |  | 2/2 |
| Days of specimen collection* (Range) | 1−3 | 4−5 | 13−24 |  | 1−2 | 3−11 |  | NA |
| HHV-6B DNA load (copies/mL) (Range) | 0−53800 | 0−29050 | 0−0 |  | 0−54850 | 0−166450 |  | 0−0 |
| *The first day of fever was defined as day 1.  LC, liquid chromatography; MS, mass spectrometry; AESD, acute encephalopathy with biphasic seizures and late reduced diffusion; cFS, complex febrile seizures; HC, healthy controls; HHV, human herpesvirus; NA, not applicable. | | | | | | | | |

| **Sup. Table 2. Patient characteristics of CSF for which LC-MS/MS was performed** | | | | | |
| --- | --- | --- | --- | --- | --- |
| **Group** | **AESD** | | |  | **cFS** |
| Phase (n) | Early (4) | Late (3) | Convalescent (3) |  | Acute (4) |
| Age (years) (Range) | 0.67−1 | 0.67−1 | 0.67−1 |  | 0.75−1 |
| Sex (Male/Female) | 2/2 | 1/2 | 1/2 |  | 2/2 |
| Days of specimen collection* (Range) | 1−3 | 4−6 | 13−30 |  | 1−2 |
| HHV-6B DNA load (copies/mL) (Range) | 0−0 | 0−0 | 0−0 |  | 0−0 |
| *The first day of fever was defined as day 1.  CSF, cerebrospinal fluid; LC, liquid chromatography; MS, mass spectrometry; AESD, acute encephalopathy with biphasic seizures and late reduced diffusion; cFS, complex febrile seizures; HHV, human herpesvirus; NA, not applicable. | | | | | |

| **Sup. Table 3. Number of differentially expressed proteins in patients with AESD early** | | | | | | | | | | | |
| --- | --- | --- | --- | --- | --- | --- | --- | --- | --- | --- | --- |
|  | vs AESD late | |  | vs AESD convalescent | |  | vs cFS acute | |  | vs HC | |
|  | Up | Down |  | Up | Down |  | Up | Down |  | Up | Down |
| Number of proteins in serum | 17 | 47 |  | 24 | 29 |  | 13 | 6 |  | 39 | 55 |
| Number of proteins in CSF | 7 | 41 |  | 11 | 47 |  | 7 | 31 |  | N/A | N/A |
| AESD, Acute encephalopathy with biphasic seizures and late reduced diffusion; cFS, complex febrile seizures; HC, healthy controls; CSF, cerebrospinal fluid. | | | | | | | | | | | |

| **Sup. Table 4.** **Differentially expressed proteins in serum from patients with AESD early compared to AESD late** | | | |
| --- | --- | --- | --- |
| **Proteins** | **Log_2_ fold change** | **Adjusted *P*-value** |  |
| Aspartate aminotransferase, cytoplasmic | 4.49828 | 0.02515 |  |
| Pleckstrin | 3.44873 | 0.01320 |  |
| Procathepsin L | 3.01562 | 0.03767 |  |
| Tropomyosin alpha-1 chain | 2.85754 | 0.00979 |  |
| Ankyrin-1 | 2.62680 | 0.01793 |  |
| Talin-1 | 2.59044 | 0.04904 |  |
| Ezrin | 2.49416 | 0.01138 |  |
| **Golgi membrane protein 1** | 2.46216 | 0.04967 |  |
| Profilin-1 | 2.33315 | 0.01767 |  |
| Neutrophil gelatinase-associated lipocalin | 2.23217 | 0.00635 |  |
| Platelet factor 4 variant | 2.15791 | 0.02090 |  |
| Alpha-actinin-1 | 1.90591 | 0.04747 |  |
| Cofilin-1 | 1.89489 | 0.01206 |  |
| Alpha-enolase | 1.86664 | 0.01063 |  |
| Triosephosphate isomerase | 1.21160 | 0.01432 |  |
| Lipopolysaccharide-binding protein | 1.10551 | 0.02073 |  |
| Collagen alpha-1(VI) chain | 0.85707 | 0.02553 |  |
| Inter-alpha-trypsin inhibitor heavy chain H4 | -0.59835 | 0.01121 |  |
| Coagulation factor XI | -0.60127 | 0.00406 |  |
| Coagulation factor V | -0.60619 | 0.01586 |  |
| Prothrombin | -0.62242 | 0.02368 |  |
| Galectin-3-binding protein | -0.63012 | 0.04277 |  |
| Clusterin | -0.63601 | 0.04783 |  |
| Complement factor I | -0.64651 | 0.00713 |  |
| Beta-1,4-glucuronyltransferase 1 | -0.66032 | 0.04381 |  |
| Complement C5 | -0.66189 | 0.00970 |  |
| Carboxypeptidase B2 | -0.66605 | 0.02354 |  |
| Complement C1q subcomponent subunit A | -0.67763 | 0.04167 |  |
| C4b-binding protein alpha chain | -0.68202 | 0.00376 |  |
| Plasminogen | -0.68208 | 0.01631 |  |
| Complement C1r subcomponent-like protein | -0.70585 | 0.02173 |  |
| Phospholipid transfer protein | -0.70833 | 0.00329 |  |
| Kallistatin | -0.71274 | 0.02844 |  |
| Vitamin K-dependent protein Z | -0.71540 | 0.02204 |  |
| Plasma protease C1 inhibitor | -0.71988 | 0.00708 |  |
| C4b-binding protein beta chain | -0.73940 | 0.00193 |  |
| Vitronectin | -0.76985 | 0.00617 |  |
| Procollagen C-endopeptidase enhancer 1 | -0.77071 | 0.04893 |  |
| Protein AMBP | -0.82136 | 0.00336 |  |
| Heparin cofactor 2 | -0.84354 | 0.02297 |  |
| Alpha-2-antiplasmin | -0.86994 | 0.00857 |  |
| Hyaluronan-binding protein 2 | -0.89672 | 0.01194 |  |
| Vitamin K-dependent protein C | -0.92676 | 0.02512 |  |
| Coagulation factor X | -0.93851 | 0.00038 |  |
| Selenoprotein P | -0.95172 | 0.02013 |  |
| Apolipoprotein E | -0.96894 | 0.01791 |  |
| Complement factor H-related protein 1 | -0.99305 | 0.03165 |  |
| Uncharacterized protein C6orf163 | -1.01068 | 0.04456 |  |
| Coagulation factor VII | -1.02241 | 0.00379 |  |
| Retinol-binding protein 4 | -1.05340 | 0.00436 |  |
| 72 kDa type IV collagenase | -1.15499 | 0.01661 |  |
| Serpin A11 | -1.18472 | 0.01261 |  |
| Fetuin-B | -1.22778 | 0.00993 |  |
| Prenylcysteine oxidase 1 | -1.23539 | 0.03374 |  |
| Complement component C9 | -1.35370 | 0.02175 |  |
| Amyloid-beta precursor protein | -1.37223 | 0.01902 |  |
| Secreted phosphoprotein 24 | -1.41680 | 0.01707 |  |
| Plasma serine protease inhibitor | -1.44967 | 0.00111 |  |
| Insulin-like growth factor II | -1.48496 | 0.01800 |  |
| Beta-Ala-His dipeptidase | -1.65401 | 0.03129 |  |
| Spectrin alpha chain, erythrocytic 1 | -1.66866 | 0.03219 |  |
| Apolipoprotein C-III | -2.34639 | 0.02939 |  |
| Apolipoprotein C-II | -3.19557 | 0.04808 |  |
| Apolipoprotein C-IV | -3.36722 | 0.00520 |  |
| **Proteins in bold** were verified by enzyme-linked immunosorbent assay. AESD, Acute encephalopathy with biphasic seizures and late reduced diffusion; cFS, complex febrile seizures; HC, healthy controls. | | | |

| **Sup. Table 5. Differentially expressed proteins in serum from patients with AESD early compared to AESD convalescent** | | |
| --- | --- | --- |
| **Proteins** | **Log_2_ fold change** | **Adjusted *P*-value** |
| DDB1- and CUL4-associated factor 4 | 5.570 | 0.002 |
| Aspartate aminotransferase, cytoplasmic | 4.634 | 0.004 |
| Cathepsin G | 4.368 | 0.038 |
| Malate dehydrogenase, cytoplasmic | 4.246 | 0.006 |
| Immunoglobulin heavy variable 1-18 | 3.655 | 0.016 |
| Myeloperoxidase | 3.645 | 0.009 |
| Ezrin | 3.619 | 0.022 |
| Histone H2A type 2-C | 3.170 | 0.028 |
| Neutrophil gelatinase-associated lipocalin | 3.063 | 0.001 |
| Immunoglobulin heavy variable 1-69 | 2.765 | 0.029 |
| Moesin | 2.764 | 0.000 |
| Spectrin beta chain, erythrocytic | 2.528 | 0.015 |
| Heat shock 70 kDa protein 1B | 2.265 | 0.033 |
| Phosphoglycerate mutase 1 | 2.218 | 0.029 |
| **Golgi membrane protein 1** | 2.194 | 0.030 |
| Lipopolysaccharide-binding protein | 2.185 | 0.002 |
| Cerebellin-1 | 2.109 | 0.006 |
| Ankyrin-1 | 2.108 | 0.045 |
| Alpha-enolase | 1.965 | 0.007 |
| Rab GDP dissociation inhibitor beta | 1.367 | 0.046 |
| von Willebrand factor | 1.245 | 0.009 |
| Periostin | 1.101 | 0.005 |
| Immunoglobulin lambda variable 3-21 | 1.058 | 0.007 |
| 14-3-3 protein zeta/delta | 0.958 | 0.002 |
| Complement C1q subcomponent subunit A | -0.598 | 0.038 |
| Prothrombin | -0.653 | 0.018 |
| Protein AMBP | -0.671 | 0.042 |
| Phospholipid transfer protein | -0.674 | 0.004 |
| Complement C1r subcomponent-like protein | -0.677 | 0.044 |
| C4b-binding protein beta chain | -0.732 | 0.010 |
| Coagulation factor XIII B chain | -0.735 | 0.024 |
| C4b-binding protein alpha chain | -0.776 | 0.011 |
| Beta-1,4-glucuronyltransferase 1 | -0.800 | 0.026 |
| Mannan-binding lectin serine protease 2 | -0.807 | 0.044 |
| Complement C5 | -0.886 | 0.007 |
| Serum amyloid A-4 protein | -0.956 | 0.043 |
| Carboxypeptidase B2 | -0.957 | 0.007 |
| Prenylcysteine oxidase 1 | -0.970 | 0.045 |
| Heparin cofactor 2 | -0.981 | 0.013 |
| Lymphocyte antigen 6H | -1.039 | 0.031 |
| Secreted phosphoprotein 24 | -1.134 | 0.021 |
| Fetuin-B | -1.151 | 0.017 |
| Complement factor H-related protein 1 | -1.195 | 0.010 |
| Plasma serine protease inhibitor | -1.403 | 0.030 |
| Spondin-1 | -1.418 | 0.043 |
| Collagen alpha-1(XVIII) chain | -1.427 | 0.022 |
| Zinc-alpha-2-glycoprotein | -1.439 | 0.020 |
| Amyloid-beta precursor protein | -1.521 | 0.005 |
| Spectrin alpha chain, erythrocytic 1 | -1.587 | 0.033 |
| Prosaposin | -1.664 | 0.044 |
| Retinol-binding protein 4 | -1.800 | 0.007 |
| Platelet factor 4 | -2.052 | 0.011 |
| Apolipoprotein C-IV | -2.649 | 0.026 |
| **Proteins in bold** were verified by enzyme-linked immunosorbent assay. AESD, Acute encephalopathy with biphasic seizures and late reduced diffusion; cFS, complex febrile seizures; HC, healthy controls. | | |

| **Sup. Table 6. Differentially expressed proteins in serum from patients with AESD early compared to healthy controls** | | |
| --- | --- | --- |
| **Proteins** | **Log_2_ fold change** | **Adjusted *P*-value** |
| C-reactive protein | 8.621 | 0.011 |
| Serum amyloid A-1 protein | 8.492 | 0.003 |
| Fructose-bisphosphate aldolase B | 4.648 | 0.003 |
| Malate dehydrogenase, cytoplasmic | 4.630 | 0.003 |
| Cathepsin G | 4.376 | 0.037 |
| Neutrophil gelatinase-associated lipocalin | 4.300 | 0.004 |
| Histone H4 | 4.269 | 0.035 |
| Myeloperoxidase | 4.139 | 0.006 |
| Histone H2A type 2-C | 4.040 | 0.012 |
| Aspartate aminotransferase, cytoplasmic | 3.898 | 0.012 |
| Procathepsin L | 3.802 | 0.008 |
| Synaptotagmin-13 | 3.548 | 0.003 |
| Phosphoglycerate mutase 1 | 3.310 | 0.003 |
| Elongation factor 1-alpha 2 | 3.022 | 0.020 |
| Histone H3.3 | 2.959 | 0.048 |
| Alpha-2-macroglobulin | 2.953 | 0.007 |
| Caspase recruitment domain-containing protein 6 | 2.952 | 0.035 |
| Poliovirus receptor | 2.863 | 0.015 |
| Rho GDP-dissociation inhibitor 2 | 2.682 | 0.032 |
| Fatty acid-binding protein, liver | 2.644 | 0.033 |
| Heat shock 70 kDa protein 1B | 2.531 | 0.015 |
| Lipopolysaccharide-binding protein | 2.452 | 0.000 |
| Golgi membrane protein 1 | 2.389 | 0.045 |
| Transketolase | 2.322 | 0.032 |
| von Willebrand factor | 2.269 | 0.000 |
| Glyceraldehyde-3-phosphate dehydrogenase | 2.235 | 0.037 |
| Immunoglobulin kappa variable 1-27 | 2.178 | 0.019 |
| Moesin | 2.114 | 0.001 |
| Leucine-rich alpha-2-glycoprotein | 1.939 | 0.004 |
| Cathepsin Z | 1.730 | 0.000 |
| Heat shock cognate 71 kDa protein | 1.633 | 0.033 |
| Triosephosphate isomerase | 1.570 | 0.022 |
| Alpha-1-acid glycoprotein 1 | 1.459 | 0.011 |
| 14-3-3 protein zeta/delta | 1.201 | 0.010 |
| Nidogen-1 | 0.977 | 0.026 |
| Alpha-1-antitrypsin | 0.872 | 0.036 |
| Lysozyme C | 0.850 | 0.037 |
| Phosphatidylinositol 3,4,5-trisphosphate 3-phosphatase and dual-specificity protein phosphatase PTEN | 0.737 | 0.032 |
| Mannosyl-oligosaccharide 1,2-alpha-mannosidase IA | 0.654 | 0.000 |
| Inter-alpha-trypsin inhibitor heavy chain H4 | -0.633 | 0.011 |
| Alpha-2-antiplasmin | -0.675 | 0.039 |
| Complement C5 | -0.679 | 0.046 |
| Coagulation factor VII | -0.687 | 0.032 |
| Vitronectin | -0.695 | 0.018 |
| Hepatocyte growth factor-like protein | -0.697 | 0.017 |
| Complement C1r subcomponent-like protein | -0.720 | 0.033 |
| Cholinesterase | -0.730 | 0.045 |
| Protein AMBP | -0.759 | 0.005 |
| Coagulation factor XIII B chain | -0.808 | 0.021 |
| Complement C1q tumor necrosis factor-related protein 3 | -0.820 | 0.009 |
| Carbonic anhydrase 2 | -0.824 | 0.003 |
| Beta-2-glycoprotein 1 | -0.833 | 0.009 |
| Heparin cofactor 2 | -0.933 | 0.020 |
| Histidine-rich glycoprotein | -0.942 | 0.044 |
| Apolipoprotein E | -0.950 | 0.035 |
| N-acetylmuramoyl-L-alanine amidase | -0.971 | 0.011 |
| Procollagen C-endopeptidase enhancer 1 | -0.971 | 0.037 |
| Inter-alpha-trypsin inhibitor heavy chain H1 | -0.984 | 0.016 |
| Phospholipid transfer protein | -0.997 | 0.000 |
| Secreted phosphoprotein 24 | -1.000 | 0.046 |
| Dematin | -1.011 | 0.000 |
| 72 kDa type IV collagenase | -1.030 | 0.022 |
| Kallistatin | -1.032 | 0.008 |
| Vitamin K-dependent protein C | -1.035 | 0.013 |
| Inter-alpha-trypsin inhibitor heavy chain H2 | -1.104 | 0.016 |
| Carboxypeptidase B2 | -1.111 | 0.002 |
| Platelet glycoprotein V | -1.118 | 0.027 |
| Superoxide dismutase [Mn], mitochondrial | -1.120 | 0.001 |
| Retinol-binding protein 4 | -1.132 | 0.015 |
| Amyloid-beta precursor protein | -1.136 | 0.035 |
| Serum amyloid A-4 protein | -1.176 | 0.021 |
| Adipocyte plasma membrane-associated protein | -1.184 | 0.040 |
| Apolipoprotein A-I | -1.380 | 0.021 |
| Leucine-rich repeat serine/threonine-protein kinase 2 | -1.412 | 0.004 |
| Mimecan | -1.435 | 0.011 |
| Collagen alpha-1(I) chain | -1.462 | 0.043 |
| Insulin-like growth factor II | -1.529 | 0.008 |
| Cholesteryl ester transfer protein | -1.574 | 0.025 |
| Selenoprotein P | -1.633 | 0.001 |
| Neurotrimin | -1.791 | 0.042 |
| Putative heat shock 70 kDa protein 7 | -1.872 | 0.020 |
| Apolipoprotein B-100 | -1.950 | 0.039 |
| Apolipoprotein C-IV | -2.002 | 0.041 |
| Single-pass membrane and coiled-coil domain-containing protein 1 | -2.047 | 0.000 |
| Prenylcysteine oxidase 1 | -2.077 | 0.001 |
| Dopamine beta-hydroxylase | -2.108 | 0.011 |
| Plasma serine protease inhibitor | -2.109 | 0.014 |
| Glucose-6-phosphate isomerase | -2.276 | 0.032 |
| Apolipoprotein A-IV | -2.426 | 0.025 |
| Apolipoprotein(a) | -2.463 | 0.049 |
| Coiled-coil alpha-helical rod protein 1 | -2.520 | 0.041 |
| Di-N-acetylchitobiase [OS=Homo sapiens] | -2.550 | 0.022 |
| Extracellular superoxide dismutase [Cu-Zn] | -3.702 | 0.035 |
| FRAS1-related extracellular matrix protein 1 | -3.916 | 0.006 |
| AESD, Acute encephalopathy with biphasic seizures and late reduced diffusion; cFS, complex febrile seizures; HC, healthy controls. | | |

| **Sup. Table 7. Differentially expressed proteins of CSF in patients with AESD early compared to AESD late** | | |
| --- | --- | --- |
| **Proteins** | **Log_2_ fold change** | **Adjusted *P*-value** |
| Single-pass membrane and coiled-coil domain-containing protein 1 | 1.305 | 0.045 |
| Immunoglobulin alpha-2 heavy chain | 1.284 | 0.008 |
| Immunoglobulin kappa variable 2-40 | 1.188 | 0.039 |
| Immunoglobulin heavy variable 3-74 | 1.106 | 0.047 |
| Immunoglobulin heavy variable 3-7 | 1.083 | 0.030 |
| Apolipoprotein A-I | 1.007 | 0.036 |
| Inter-alpha-trypsin inhibitor heavy chain H2 | 0.772 | 0.001 |
| Hepatitis A virus cellular receptor 2 | -0.645 | 0.014 |
| Mannan-binding lectin serine protease 2 | -0.645 | 0.014 |
| Phosphatidylethanolamine-binding protein 1 | -0.736 | 0.024 |
| L-lactate dehydrogenase B chain | -0.810 | 0.038 |
| Prosaposin | -0.860 | 0.034 |
| Complement C1r subcomponent | -0.914 | 0.026 |
| Complement C1q subcomponent subunit C | -0.952 | 0.020 |
| Complement C2 | -1.021 | 0.024 |
| Metallothionein-3 | -1.031 | 0.001 |
| Neuromodulin | -1.037 | 0.030 |
| Guanine deaminase | -1.048 | 0.034 |
| Complement C1q subcomponent subunit B | -1.050 | 0.028 |
| L-lactate dehydrogenase A chain | -1.060 | 0.046 |
| Fructose-bisphosphate aldolase A | -1.087 | 0.045 |
| Beta-2-microglobulin | -1.223 | 0.015 |
| Fructose-bisphosphate aldolase C | -1.307 | 0.009 |
| Phosphoglycerate mutase 1 | -1.307 | 0.000 |
| Complement component C9 | -1.319 | 0.048 |
| Superoxide dismutase [Mn], mitochondrial | -1.432 | 0.036 |
| Ankyrin-1 | -1.469 | 0.021 |
| Keratin, type I cytoskeletal 14 | -1.501 | 0.014 |
| Low affinity immunoglobulin gamma Fc region receptor III-A | -1.555 | 0.016 |
| Peptidyl-prolyl cis-trans isomerase A | -1.673 | 0.035 |
| Osteopontin | -1.746 | 0.018 |
| Chitinase-3-like protein 1 | -2.033 | 0.042 |
| Monocyte differentiation antigen CD14 | -2.182 | 0.005 |
| Phosphoglycerate kinase 1 | -2.512 | 0.027 |
| Parathymosin | -2.557 | 0.049 |
| Protein-L-isoaspartate(D-aspartate) O-methyltransferase | -2.775 | 0.011 |
| 14-3-3 protein epsilon | -2.878 | 0.024 |
| Endoplasmic reticulum chaperone BiP | -2.978 | 0.028 |
| Ubiquitin carboxyl-terminal hydrolase isozyme L1 | -2.983 | 0.032 |
| 14-3-3 protein eta | -3.077 | 0.001 |
| Gamma-enolase | -3.126 | 0.029 |
| Paralemmin-1 | -3.382 | 0.031 |
| Neurogranin | -3.569 | 0.028 |
| Glyceraldehyde-3-phosphate dehydrogenase | -3.628 | 0.004 |
| Cofilin-1 | -3.904 | 0.033 |
| 14-3-3 protein zeta/delta | -4.044 | 0.007 |
| 14-3-3 protein gamma | -4.622 | 0.006 |
| AESD, Acute encephalopathy with biphasic seizures and late reduced diffusion; cFS, complex febrile seizures; HC, healthy controls. | | |

| **Sup. Table 8. Differentially expressed proteins of CSF in patients with AESD early compared to AESD convalescent** | | |
| --- | --- | --- |
| **Proteins** | **Log_2_ fold change** | **Adjusted *P*-value** |
| Serum amyloid A-1 protein [OS=Homo sapiens] | 1.305 | 0.045 |
| Single-pass membrane and coiled-coil domain-containing protein 1 [OS=Homo sapiens] | 1.284 | 0.008 |
| Vacuolar protein sorting-associated protein 4A [OS=Homo sapiens] | 1.188 | 0.039 |
| Caspase recruitment domain-containing protein 6 [OS=Homo sapiens] | 1.106 | 0.047 |
| Sex hormone-binding globulin [OS=Homo sapiens] | 1.083 | 0.030 |
| Platelet glycoprotein V [OS=Homo sapiens] | 1.007 | 0.036 |
| Pleckstrin [OS=Homo sapiens] | 0.772 | 0.001 |
| 7-methylguanosine phosphate-specific 5'-nucleotidase [OS=Homo sapiens] | -0.645 | 0.014 |
| Immunoglobulin superfamily containing leucine-rich repeat protein [OS=Homo sapiens] | -0.645 | 0.014 |
| Probable G-protein coupled receptor 158 [OS=Homo sapiens] | -0.736 | 0.024 |
| Nidogen-2 [OS=Homo sapiens] | -0.810 | 0.038 |
| Afamin [OS=Homo sapiens] | -0.860 | 0.034 |
| Apolipoprotein E [OS=Homo sapiens] | -0.914 | 0.026 |
| Alpha-1-antichymotrypsin [OS=Homo sapiens] | -0.952 | 0.020 |
| Mannose-binding protein C [OS=Homo sapiens] | -1.021 | 0.024 |
| Prosaposin [OS=Homo sapiens] | -1.031 | 0.001 |
| Retinol-binding protein 4 [OS=Homo sapiens] | -1.037 | 0.030 |
| Thymosin beta-4 [OS=Homo sapiens] | -1.048 | 0.034 |
| Monocyte differentiation antigen CD14 [OS=Homo sapiens] | -1.050 | 0.028 |
| L-lactate dehydrogenase B chain [OS=Homo sapiens] | -1.060 | 0.046 |
| Tyrosine-protein kinase receptor UFO [OS=Homo sapiens] | -1.087 | 0.045 |
| Immunoglobulin heavy variable 6-1 [OS=Homo sapiens] | -1.223 | 0.015 |
| Phosphatidylethanolamine-binding protein 1 [OS=Homo sapiens] | -1.307 | 0.009 |
| Neuromodulin [OS=Homo sapiens] | -1.307 | 0.000 |
| Osteopontin [OS=Homo sapiens] | -1.319 | 0.048 |
| Parkinson disease protein 7 [OS=Homo sapiens] | -1.432 | 0.036 |
| 14-3-3 protein eta [OS=Homo sapiens] | -1.469 | 0.021 |
| Coactosin-like protein [OS=Homo sapiens] | -1.501 | 0.014 |
| Superoxide dismutase [Mn], mitochondrial [OS=Homo sapiens] | -1.555 | 0.016 |
| Fructose-bisphosphate aldolase C [OS=Homo sapiens] | -1.673 | 0.035 |
| Gamma-glutamyl hydrolase [OS=Homo sapiens] | -1.746 | 0.018 |
| Phosphoglycerate mutase 1 [OS=Homo sapiens] | -2.033 | 0.042 |
| Aspartate aminotransferase, cytoplasmic [OS=Homo sapiens] | -2.182 | 0.005 |
| L-lactate dehydrogenase A chain [OS=Homo sapiens] | -2.512 | 0.027 |
| Flotillin-1 [OS=Homo sapiens] | -2.557 | 0.049 |
| SH3 domain-binding glutamic acid-rich-like protein 3 [OS=Homo sapiens] | -2.775 | 0.011 |
| Ankyrin-1 [OS=Homo sapiens] | -2.878 | 0.024 |
| Ubiquitin carboxyl-terminal hydrolase isozyme L1 [OS=Homo sapiens] | -2.978 | 0.028 |
| Glucose-6-phosphate isomerase [OS=Homo sapiens] | -2.983 | 0.032 |
| Phosphoglycerate kinase 1 [OS=Homo sapiens] | -3.077 | 0.001 |
| 14-3-3 protein epsilon [OS=Homo sapiens] | -3.126 | 0.029 |
| Plasma serine protease inhibitor [OS=Homo sapiens] | -3.382 | 0.031 |
| Gamma-enolase [OS=Homo sapiens] | -3.569 | 0.028 |
| Fetuin-B [OS=Homo sapiens] | -3.628 | 0.004 |
| Protein Z-dependent protease inhibitor [OS=Homo sapiens] | -3.904 | 0.033 |
| Protein kinase C and casein kinase substrate in neurons protein 1 [OS=Homo sapiens] | -4.044 | 0.007 |
| Neurofilament medium polypeptide [OS=Homo sapiens] | -4.622 | 0.006 |
| Coagulation factor IX [OS=Homo sapiens] |  |  |
| Glyceraldehyde-3-phosphate dehydrogenase [OS=Homo sapiens] |  |  |
| Microtubule-associated protein 2 [OS=Homo sapiens] |  |  |
| Chitinase-3-like protein 1 [OS=Homo sapiens] |  |  |
| Rab GDP dissociation inhibitor beta [OS=Homo sapiens] |  |  |
| Ubiquitin-60S ribosomal protein L40 [OS=Homo sapiens] |  |  |
| Paralemmin-1 [OS=Homo sapiens] |  |  |
| Keratin, type I cytoskeletal 14 [OS=Homo sapiens] |  |  |
| Fatty acid-binding protein, heart [OS=Homo sapiens] |  |  |
| 14-3-3 protein zeta/delta [OS=Homo sapiens] |  |  |
| 14-3-3 protein gamma [OS=Homo sapiens] |  |  |
| AESD, Acute encephalopathy with biphasic seizures and late reduced diffusion; cFS, complex febrile seizures; HC, healthy controls. | | |
